# Iron and the Pathophysiology of Diabetes

**DOI:** 10.1146/annurev-physiol-022522-102832

**Published:** 2022-09-22

**Authors:** Alexandria V Harrison, Felipe Ramos Lorenzo, Donald A. McClain

**Affiliations:** 1 Department of Internal Medicine, Wake Forest University School of Medicine, Winston-Salem, North Carolina, USA; 2 Department of Veterans Affairs, W.G. (Bill) Hefner Veterans Affairs Medical Center, Salisbury, North Carolina, USA

**Keywords:** diabetes, iron, hepcidin, hemochromatosis, adipocyte, insulin

## Abstract

High iron is a risk factor for type 2 diabetes mellitus (T2DM) and affects most of its cardinal features: decreased insulin secretion, insulin resistance, and increased hepatic gluconeogenesis. This is true across the normal range of tissue iron levels and in pathologic iron overload. Because of iron’s central role in metabolic processes (e.g., fuel oxidation) and metabolic regulation (e.g., hypoxia sensing), iron levels participate in determining metabolic rates, gluconeogenesis, fuel choice, insulin action, and adipocyte phenotype. The risk of diabetes related to iron is evident in most or all tissues that determine diabetes phenotypes, with the adipocyte, beta cell, and liver playing central roles. Molecular mechanisms for these effects are diverse, although there may be integrative pathways at play. Elucidating these pathways has implications not only for diabetes prevention and treatment, but also for the pathogenesis of other diseases that are, like T2DM, associated with aging, nutrition, and iron.

## 1. INTRODUCTION

Macronutrients play an undisputed role in conferring an increased risk of type 2 diabetes mellitus (T2DM), but the micronutrient iron is also a risk determinant, not only in pathologic excess or deficiency, but also within its very broad range of normal levels. This review addresses the evidence for a connection between iron and diabetes risk, first providing a brief review of iron homeostasis and the evidence linking tissue iron levels with diabetes in humans. Because in many cases the aforementioned topics have been the subject of detailed reviews, this review concentrates on mechanistic studies addressing how iron regulates metabolism in the principal tissues that determine glucose homeostasis. We hope to support the overarching hypothesis that the central importance of iron availability to fuel metabolism and mitochondrial function has resulted in a tight coupling of iron availability to metabolic regulation. Not surprisingly, the role of iron extends to most aspects of metabolism—lipid, protein, nucleic acid, and carbohydrate—but this review concentrates on the regulation of glucose homeostasis. Finally, although iron is also implicated in many diabetic complications (e.g., 1), we focus on the role of iron in the pathogenesis of diabetes itself.

## 2. OVERVIEW OF IRON METABOLISM

**Figure 1:**
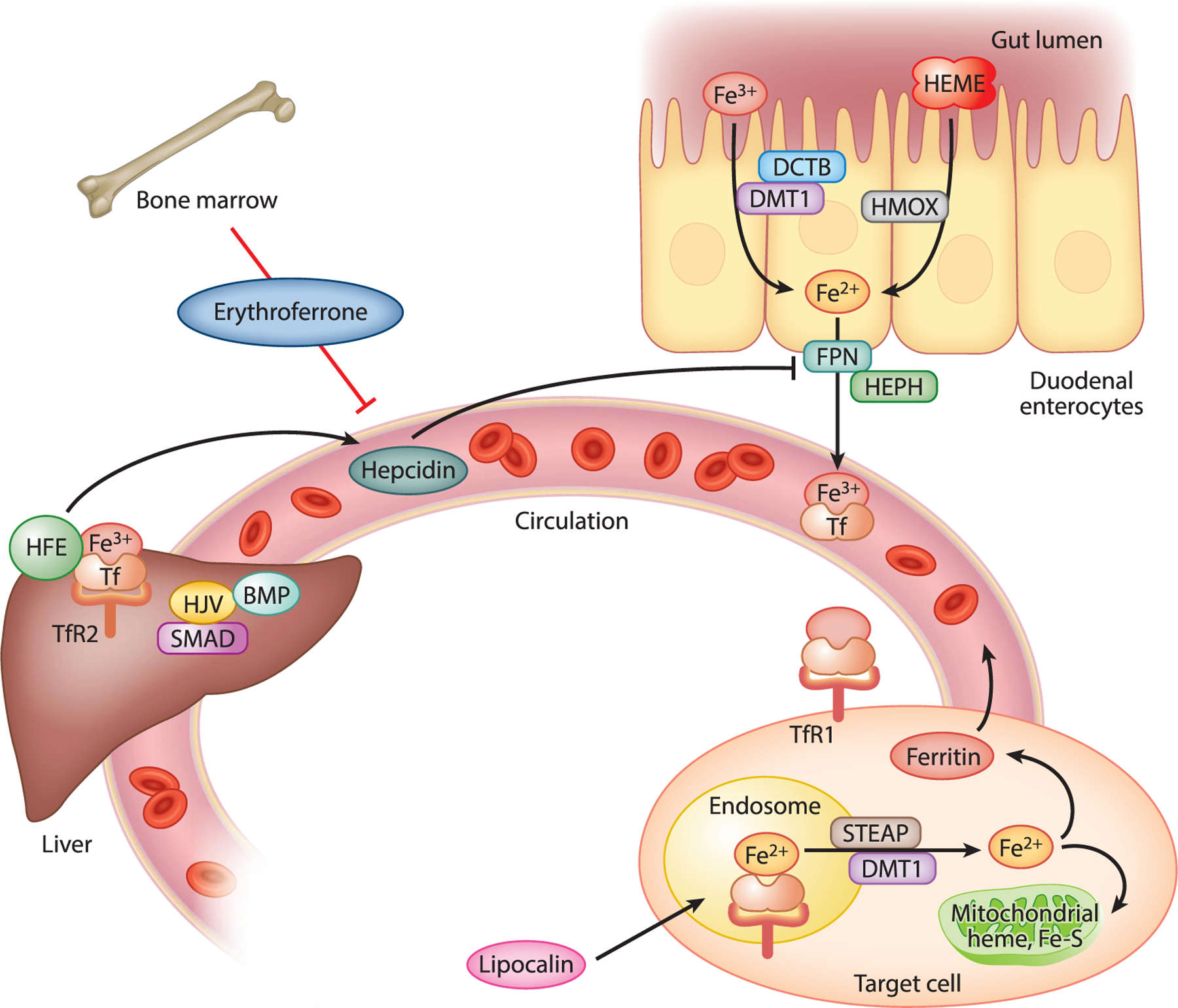
Iron homeostasis. Intestinal free ferric (Fe^3+^) iron is reduced to Fe^2+^ by duodenal cytochrome B (DCTB) and enters the cell through the divalent metal transporter 1 (DMT1). Dietary heme is directly absorbed, and iron is released by heme oxygenase (HMOX). Iron exits the enterocyte through the export channel ferroportin (FPN). After oxidation by hephaestin (HEPH), iron binds to transferrin (Tf) in the circulation, which binds to transferrin receptors (TfR) 1 and 2 on target cells. Iron is released from TfRl after acidification in the endosome, reduced by six-transmembrane epithelial antigen of the prostate (STEAP), and enters the cytosol through DMT1 where it is used (e.g., for heme or Fe-S-cluster synthesis). Excess iron is sequestered by ferritin. Apo-ferritin secreted into the circulation serves as a marker for tissue iron stores. In the fiver, Tf binds TfR2 and the protein HFE and signals the production of hepcidin via hemojuvelin (HJV), bone morphogenic protein (BMP), and the SMAD signal transduction pathway. Hepcidin induces downregulation of FPN, thus acting as a negative feedback regulator of further intestinal iron absorption. Figure adapted with permission from Reference 23.

Early organisms evolved to take advantage of iron for metabolic and other redox reactions. In parallel, there also evolved an important link between iron availability and metabolism. Fuel choice in yeast, for example, is tighdy and reciprocally coupled to iron (2), and many of those mechanisms are conserved in humans. While necessary for metabolism, iron is also a potent oxidant, and complex systems to regulate its uptake, storage, and bioavailability also evolved (3,4) (Figure 1). Some key components of those systems include:

- Regulated iron entry into the bloodstream from the gut through the iron export channel ferroportin (SLC40A1);
- Binding of iron in the circulation to transferrin and endocytic uptake into cells by the transferrin receptor;
- An independent uptake pathway mediated by lipocalin 2, especially important in early development;
- Secretion by the liver of the peptide hepcidin in response to sensing of transferrin-bound iron, leading to negative feedback regulation of ferroportin;
- A rapid intracellular response system to rising iron mediated by iron regulatory proteins (IRPs) bound to the messenger RNAs (mRNAs) of key proteins. Free iron releases the IRPs from the mRNAs, posttranscriptionally downregulating the transferrin receptor mRNA to block further iron uptake and initiating translation of the iron storage protein ferritin that sequesters excess iron; and
- An autophagic mechanism (ferritinophagy) to mobilize ferritin-bound iron in situations of iron deficiency.

Other components—the bone marrow-derived erythroferrone, a large number of intracellular chaperones, and mechanisms for incorporation of iron into heme- and iron-sulfur-complexes—add layers of complexity and regulation.

While much of the regulatory system serves to protect from iron excess, after the agrarian revolution, iron deficiency also became a significant problem for human health and is today the most common nutritional deficiency in developing countries (5). In the face of this more recent pressure, the most common human mutations affecting iron homeostasis increase iron availability by damping the iron regulatory system. For example, mutations of HFE, a protein involved in triggering hepcidin release, were adaptive 10–20,000 years ago in areas of Europe where dietary iron intake was low, but they cause iron overload [hereditary hemochromatosis (HH)] when intake is higher. Anemia secondary to inadequate dietary iron and conditions such as hookworm infection led to the iron fortification of foods in the 1940s. Combined with increased red meat consumption—heme iron is absorbed ~2 0-fold more efficiendy than elemental iron—iron excess now exists in a substantial portion of the US population (6).

High tissue iron does not come without cost. We review the causal association of high iron with diabetes, but increased iron stores are associated with numerous diseases that also share aging and ovemutrition as risk factors. For example:

- High iron is associated with not only T2DM but also nonalcoholic steatohepatitis (NASH) (7), Parkinson’s and Alzheimer’s diseases (8), colon/breast cancer (9), and cardiovascular disease (10).
- Telomere length, a biomarker of many age-related diseases, is inversely related to tissue iron (11).
- Brain iron is among the best predictors of *τ*-protein tangles and cognitive decline in Alzheimer’s disease (12).
- Metformin, an antidiabetic drug also being tested as an antiaging (13) and anticancer agent, was recendy demonstrated in yeast to primarily induce an iron starvation response (14).

## 3. IRON AND DIABETES RISK

The dangers of excess iron were first recognized in conditions of pathologic iron overload such as HH, which is associated with diabetes, NASH, cardiomyopathy, and other complications (15). The key pathogenic mechanism for diabetes in HH is insulin deficiency (16), and a causal role for iron has been demonstrated by improvements in insulin secretion after iron reduction by phlebotomy (17). Increased risk of diabetes is also seen with transfusional iron overload in conditions, including beta thalassemia (18, 19), and post-bone marrow transplant (20). Similar to HH, in thalassemia, aggressive iron chelation therapy is associated with a reduction in the incidence of diabetes (19). Important differences in disease pathophysiology exist among these conditions, at least partly related to the fact that HH is a low-hepcidin state, whereas transfusional iron overload (or dietary excess) is characterized by high hepcidin. High hepcidin will downregulate the export channel ferroportin, causing any cell or tissue with significant ferroportin expression to be iron overloaded. In iron overload resulting from low hepcidin states, such as HH, in contrast, the same cells will be paradoxically iron underloaded with low hepcidin due to the failure to downregulate ferroportin. Among the cells with high ferroportin that contribute to diabetes pathogenesis are macrophages and adipocytes, and both play roles in the effects of iron on metabolic regulation that are quite different between HH and dietary iron overload. Iron overload from transfusions is, like HH, associated with insulin deficiency, but unlike HH and because of high hepcidin and resultant high iron in adipocytes and macrophages, transfusional overload is also associated with another canonical feature of T2DM, insulin resistance (see 18 and below).

Recently it has become clear that iron is also a risk factor for typical T2DM, a risk that increases progressively in the general population through the entire normal range of serum ferritin, a marker of tissue iron stores (21,22). This association has been reviewed extensively (23,24) and confirmed in a recent meta-analysis (25). The risk is manifest in multiple races and ethnicities and also seen in gestational diabetes and prediabetes. Like pathologic iron overload, a causal relationship between iron and diabetes is shown by the facts that high-normal iron is sufficient to cause diabetes in rodents (26) and, in small studies, iron reduction has been shown to improve glycemia in human T2DM (27–31).

Further details on individual studies linking diabetes and iron epidemiologically can be accessed through the reviews cited above (21–25), but this review focuses on tissue-specific aspects of disease pathogenesis. The use of ferritin as a marker for tissue iron by many of these studies, however, does merit comment. Ferritin can also be a marker of systemic inflammation, and given the role of inflammation in obesity and T2DM, the question arises whether ferritin is primarily reflecting inflammation or tissue iron. It is true that in cases of extreme inflammation like sepsis, or when iron levels are undergoing large fluxes such as in iron reduction therapy, ferritin may temporarily lose its coupling to tissue iron levels. Nevertheless, ferritin is a reliable surrogate for tissue iron in the general population. In one of the largest studies linking ferritin to diabetes risk, high ferritin was accounted for by increased dietary iron intake and was seen in the absence of elevations in markers of inflammation (32). When iron is assessed by more specific measures (e.g., biopsy or magnetic resonance imaging), even in the setting of inflammatory states such as sickle cell disease and biopsy-proven NASH, ferritin still mainly reflects iron stores rather than inflammation (33–37). The contribution of obesity per se to serum ferritin levels is likewise only a small percentage of the large normal range of ferritin (38).

## 4. IRON AND OBESITY

Obesity is one of the major risk factors for T2DM; thus, the effects of iron on weight, independent of diabetes, are important to consider. The specific effects of iron on adipocyte biology and adipokines such as leptin are discussed below. At the level of the intact organism, however, iron deficiency (defined as iron levels being low enough to cause anemia) is associated with obesity in children (39). This is consistent with the effects of low iron to increase leptin and therefore appetite, but other effects of iron on overall weight and metabolism are more complex and multifactorial. Infants with iron deficiency are, for example, hypometabolic (40), an effect that is consistent with the hypermetabolic state and relative protection from obesity afforded by high tissue iron (41–43). Although these phenomena would suggest a diabetes protective effect of high iron, they do not factor in the deleterious effects of iron on insulin secretory capacity nor the fact that it is still possible to “eat one’s way through” this relative protection. In studies of HH, for example, it has been noted that individuals with high iron and overweight are especially prone to diabetes, presumably because their relative insulin deficiency cannot compensate for the insulin resistance of obesity, as well as in individuals with normal insulin reserves (16). Furthermore, some of these effects may be specific to childhood obesity and to overt iron deficiency as opposed to low-normal iron levels.

Another level of complexity is that obesity can also affect iron homeostasis. For example, a study of obese children revealed high levels of hepcidin, possibly triggered by higher levels of inflammatory cytokines (44). Thus, there may be a vicious cycle at work wherein iron deficiency contributes to hyperphagia and hypometabolism, but also to high hepcidin that would tend to worsen the iron deficiency. The effects of obesity on the absorption and tissue handling of iron are, like those of iron on obesity, complex and multifactorial (45). In addition, diets that are poor in iron may also be less healthy in general. Thus, there are numerous situations wherein causal connections among the factors linking iron, obesity, and diabetes risk may be bidirectional, emphasizing the need for controlled studies wherein single variables can be manipulated. But in at least some such studies, the ability of a high-fat diet to cause insulin resistance and subsequently change iron handling has been validated (46).

## 5. ORGAN-SPECIFIC MECHANISMS UNDERLYING THE CONTRIBUTION OF IRON TO DIABETES

**Figure 2:**
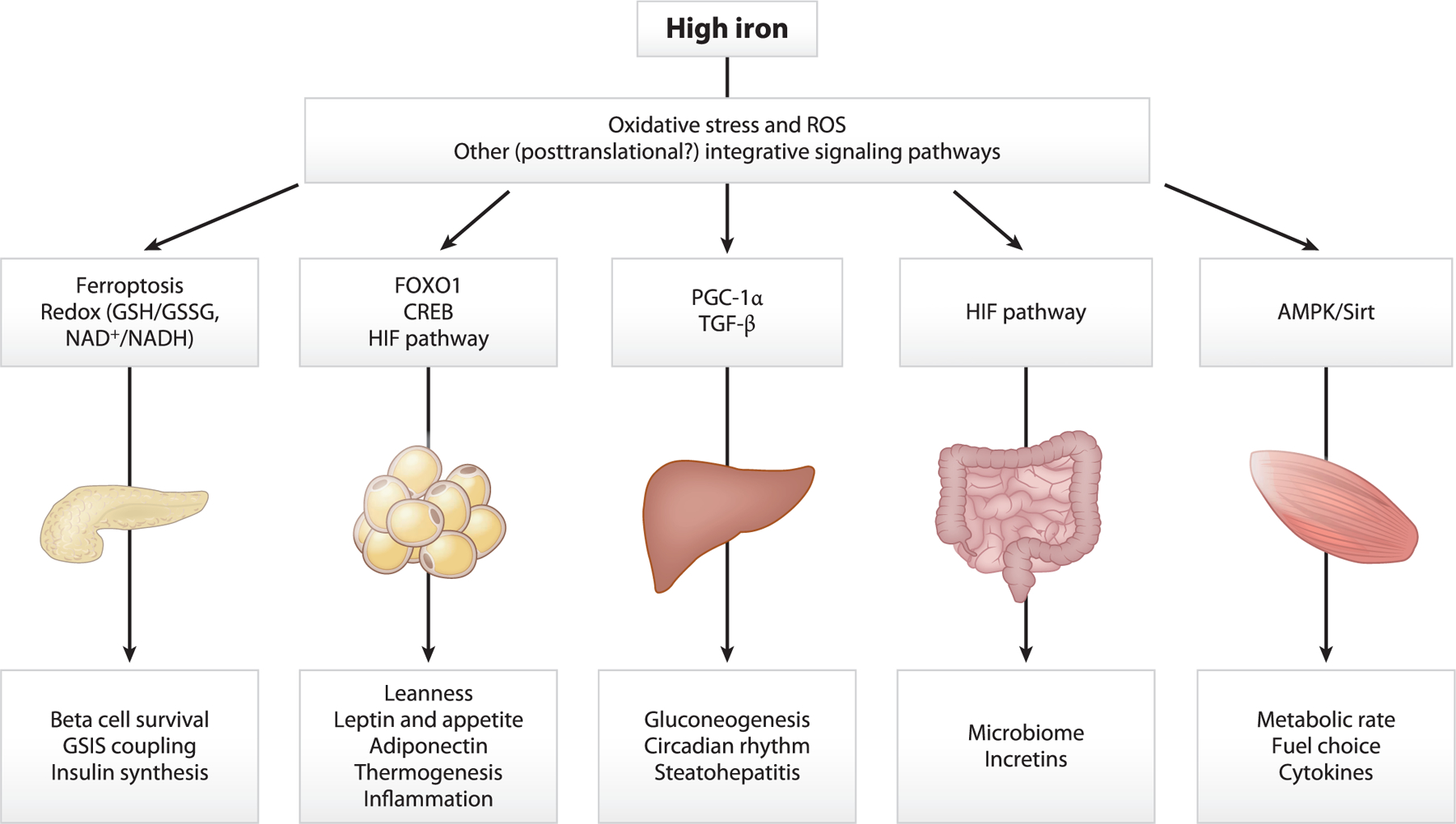
Select effects of high iron on organ systems relevant to glucose homeostasis and diabetes pathogenesis. Demonstrated mechanisms for effects are shown above and cellular/endocrine/molecular effects below. In some cases, iron deficiency has the opposite effect of high iron, for example, contributing to obesity as opposed to leanness. In other cases, for example, insulin secretion where iron is required by beta cells but excess iron may become toxic, iron deficiency has similar effects compared to high iron. Abbreviations: AMPK, AMP-dependent kinase; CREB, cAMP response element-binding protein; FOXOl, forkhead box Ol; GSH/GSSG, glutathione and glutathione disulfide; GSIS, glucose-stimulated insulin secretion; HIF, hypoxia-inducible factor; NAD+, oxidized nicotinamide adenine dinucleotide; NADH, reduced nicotinamide adenine dinucleotide; PGC-lα, peroxisome proliferator-activated receptor γ coactivator lα; ROS, reactive oxygen species; TGF-β, transforming growth factor β.

Consistent with the fundamental roles of iron in so many aspects of metabolism, mechanistic studies have revealed numerous pathways through which iron regulates fuel homeostasis, weight, appetite regulation, and hormone secretion. Some of these effects, as well as elements of the signal transduction pathways mediating them, are shown in Figure 2. Many of these have been elucidated in animal models of pathological iron overload and in wild-type mice on different levels of dietary iron. The latter models were developed to represent the very broad range of normal tissue iron levels in normal humans as well as overt iron deficiency. Our group has, for example, used mice on diets containing 4 mg, 35 mg, and 2,000 mg of elemental iron per kilogram of chow (47). If mice after weaning are initially fed a diet of normal iron (usually 35 mg/kg) for 2 months before starting the defined diets, the 4 mg/kg diet allows normal fertility and maintenance of normal blood hemoglobin concentrations for at least 8 months of life. Mice fed the 4 mg/kg diet at weaning, however, become anemic and are a model of iron deficiency. Thirty-five mg/kg iron is a good approximation of the diet of a mouse in the wild. Mice on 2,000 mg/kg show time-dependent higher tissue iron levels, and after a few months on the high iron diet hepatic iron stores are ~4 times those seen in the mice on the low-normal iron diets, well within the ~ 10-fold range of hepatic iron seen in healthy humans. In considering animal model data, it should be remembered that:

- Accumulation of dietary iron in tissues increases over time in the higher-iron diets. More rapid iron overload can be achieved with parenteral infusions, but these methods carry with them potential complications.
- Accumulation of iron is not linear with the levels in the chow because of negative feedback provided by hepcidin.
- Iron parameters differ in different strains of mice.

Thus, it is important consider the details of diet, strain, and age when interpreting animal data. Standards for the degree of iron overload should be established, as determined by not only serum ferritin but also actual measures of tissue (e.g., liver) iron and cellular reporters of iron status such as mRNA levels of the transferrin receptor that are directly regulated by bioavailable iron via IRPs.

### 5.1. Iron and Insulin Secretion

Loss of insulin secretion is a hallmark of all common forms of diabetes and also associated with iron-related diabetes resulting from either pathologic iron overload (16, 17, 19) or dietary excess (26). As is the case especially for T2DM, multiple mechanisms contribute to this loss of insulin secretion.

#### 5.1.1. Excess iron and beta cell survival.

One major contributor to the loss of insulin secretory capacity in diabetes caused by high iron is oxidative stress. Because of the fine-tuning of the redox state required for normal insulin secretion (48), the beta cell is particularly sensitive to reactive oxygen species (ROS). A canonical pathway for iron-induced cell damage is the generation of free radicals and other ROS from peroxides (Fenton chemistry), but the processes of iron toxicity in islets are more numerous, complex, and nuanced. One of the principal antioxidant-protective enzymes in pancreatic beta cells is glutathione peroxidase 4 (GPx4), and its activity in reducing lipid peroxides plays a particular role in protection from iron-induced damage and death in those cells. Thus, overexpression of GPx4 can rescue islets from stress induced by high iron and high glucose (49). This may play a particularly important role in both T2DM and iron overload, wherein GSH (the substrate oxidized in the GPx4 reaction) and GPx4 are diminished (50).

In addition to its antioxidant role, GSH is also central to iron trafficking and many iron-dependent pathways such as iron-sulfur cluster synthesis (51) and triggering ferroptosis (52), an iron-dependent noncaspase-mediated cell death pathway that involves GSH-dependent GPx and lipid oxidation. Ferroptosis plays an important role in cellular senescence (53), a significant pathway of beta cell loss in diabetes (54). Iron and ferroptosis also participate in beta cell loss secondary to glucolipotoxicity (55), and inhibition of ferroptosis (56) can rescue islets from stress induced by high iron and high glucose. All of these mechanisms likely contribute to the finding that iron chelation improves islet survival in transplantation models (reviewed in 57).

There are likely other reasons for the particular sensitivity of beta cells to high iron. Beta cells express high levels of the nonspecific divalent metal transporter 1 (DMT1) (58), required for entry into the cytosol of not only iron but also other metals, including zinc, which beta cells require for insulin packaging in secretory granules. Although most cellular iron uptake is mediated by the transferrin receptor, iron can be taken up directly by DMT1. This process would be accentuated in pathologic iron overload with high transferrin saturation and resultant labile or nontransferrin-bound iron. DMT1 is also upregulated by inflammatory cytokines (59), which could further accentuate that process. Even under normal iron conditions mouse beta cells lacking DMT1 are protected against damage from inflammatory cytokines and in both type 1 and type 2 diabetes models (60). Beta cells lacking DMT1 also exhibit defective glucose-stimulated insulin secretion, implying a physiological role for DMT1, but whether that is related to iron or to entry of other metals (e.g., zinc or manganese for metallation of superoxide dismutase 2) is not known.

The exocrine pancreas also accumulates iron, so damage to beta cells through their proximity to inflamed acinar cells has also been posited as a potential mechanism for resultant diabetes (61). Those authors, in fact, questioned whether iron had any effects on islets, stating that histochemical staining of islets of mice lacking hepcidin did not reveal excess iron and that those mice did not have impaired glucose tolerance. In fact, however, their data show a phenotype very similar to and consistent with the low hepcidin state. Namely, mice that lack the HFE protein commonly mutated in HH exhibit enhanced insulin sensitivity and improved glucose tolerance until the loss of beta cells reaches a critical level (62), as discussed in Section 3. The latter study found high iron in islets in the HFE mutant mice but by using a more sensitive methodology.

#### 5.1.2. Iron and coupling of glucose metabolism to insulin secretion.

Although loss of insulin secretory capacity is certainly related to the loss of beta cell mass induced by high iron, there is also evidence that coupling of insulin secretion to increased glucose levels is also influenced by iron. Thus, the mouse model of HH is characterized by not only decreased insulin secretory capacity but also a rightward shift in the glucose-insulin secretion dose-response curve (62). While diminished mitochondrial glucose oxidative capacity likely plays a role, other factors are also at work. GSH, whose levels are low in diabetes, is also important for coupling glucose to the stimulation of insulin secretion through the isocitrate-to-SUMO-specific peptidase 1 pathway (63). Another potential link of this pathway to iron is the fact that cytosolic aconitase, which converts citrate to isocitrate, is iron sensitive and is also one of the iron regulatory proteins (IRP-1) responsible for iron sensing (64).

#### 5.1.3. Iron deficiency and insulin secretion.

Iron deficiency can also impair insulin secretion, as demonstrated in mice with deletion of DMT1 (65). This is not surprising given the importance of iron to mitochondrial metabolism and its requirement for normal glucose-stimulated insulin secretion. Although a plausible explanation is the insufficient metalation of mitochondrial proteins required for glucose oxidation and stimulation of insulin secretion, other pathways are also involved. Leibold and colleagues (66), for example, have shown that loss of the iron-regulatory protein IRP-2 results in functional iron deficiency in beta cells. This, in turn, results in insulin deficiency from abnormal transfer RNA (tRNA) processing, leading to reduced synthesis and processing of proinsulin. Given the breadth of cellular processes that require and are regulated by iron, it is likely that many other pathways will also be found to be involved.

#### 5.1.4. Iron and type 1 diabetes.

In addition to the work focusing on T2DM, studies have also associated the development of type 1 diabetes with dietary iron intake (67). Fewer mechanistic studies have been done in this arena. For example, might the immune system be as much a factor as beta cell survival in this association (48)?

### 5.2. Iron and the Adipocyte

Adipocyte health is required for normal metabolic regulation in all of the organs involved in energy homeostasis, so it is not surprising that adipose tissue plays a crucial role in the pathogenesis of T2DM (68, 69). There are multiple pathways through which this is hypothesized to occur. For example, adipocyte tissue functions as an endocrine organ producing key hormones such as leptin (70) and adiponectin (71), whose key roles in metabolic regulation have been recendy reviewed (72, 73). The role of iron in adipocyte function, however, has only been recognized relatively recently.

#### 5.2.1. Adipose tissue as an iron sensor.

Among the first discoveries about the role of iron in adipocyte biology was that high iron is an important negative regulator of both leptin (74) and adiponectin (30). These findings are therefore also consistent with a role for iron in integrating the macro- and micronutrient status of the organism with overall metabolic regulation, for which leptin and adiponectin are crucial. The physiological relevance of this regulation has been demonstrated by, for example, leptin-dependent changes in feeding behavior (74) and adiponectin-dependent increases in serum triglycerides (75) in mice exposed to higher yet nonpathologic levels of iron. To achieve this function, several genes involved in iron metabolism that are otherwise restricted in their expression, including ferroportin, hemojuvelin, transferrin, the HFE human hemochromatosis protein, and hepcidin, are expressed in adipose tissue (30, 76–78).

#### 5.2.2. High adipose tissue iron increases diabetes risk.

A possible relationship between adipocyte iron and the antidiabetic adipoldne adiponectin was suggested by high adiponectin levels in individuals with iron overload from hereditary hemochromatosis and low adiponectin levels in individuals with dietary iron overload (30). This apparent paradox was shown to be because adipocytes express the iron export channel ferroportin. In low hepcidin states such as hereditary hemochromatosis the channel is not downregulated, allowing iron egress from cells and paradoxical iron underloading even in the face of systemic iron overload. In dietary iron excess, by contrast, hepcidin is high, ferroportin is downregulated, and adipocyte iron levels are high. Direct regulation of adiponectin by iron was demonstrated in cell and animal models (30). The direct role of adipocyte iron in these processes was demonstrated by adipocyte-specific loss of ferroportin that resulted in adipocyte iron loading, decreased adiponectin, and insulin resistance (30).

The findings discussed above were challenged in later work showing that ferroportin deletion in adipocytes did not affect adipocyte iron levels, adiponectin, or insulin sensitivity (79). The reasons for the different findings are not clear, although in the latter work, mice were not exposed to higher-iron diets, so it may be the case that iron overloading was not achieved even in the ferroportin knockout mice. Modulation of adipocyte iron through manipulation of ferroportin, however, has been confirmed by other investigators (80). Likewise, the negative correlation of adiponectin with markers of both total body iron stores (75,81,82) and adipocyte iron specifically (83, 84) has been replicated in several laboratories.

The link of insulin resistance and low adiponectin levels with adipocyte iron was also demonstrated in a novel model of overexpression of mitochondrial ferritin (FtMT) (85). This results in adipocyte iron overload, evidence of increased adipocyte ROS, low adiponectin, insulin resistance, and glucose intolerance despite protection from diet-induced obesity. This model differs from those above insofar as ferritin should sequester the iron and make it nonbioavailable, although cytosolic iron also increases, suggesting more global changes in iron homeostasis. Thus, whether some of the observed phenotypes—increased ROS and decreased mitochondrial respiration, for example—are actually due to changes in fluxes, partitioning, or even deficiency of iron in select pools is not clear.

#### 5.2.3. Other inflammatory adipokines.

In addition to the modulation by iron of adiponectin and leptin—important regulators in themselves of metabolism, appetite, and insulin signaling—iron also regulates inflammatory cytokines produced by adipocytes. In cultured 3T3-L1 adipocytes, for example, iron increases interleukin 6 (IL-6) and tumor necrosis factor α (TNF-α) mRNA (86). Interestingly, iron accumulation in the absence of increased dietary exposure is seen in adipocytes in *ob/ob* (*lep*^−/−^) mice, consistent with the effects of obesity on iron handling described in Section 4 (87). This is associated with increases in IL-6 and TNF-α, but these levels normalize with iron chelation treatment, demonstrating that, although obesity may affect iron, iron is nevertheless the mediator that plays a direct and causal role in modulation of these proinflammatory and prodiabetic factors. Iron induces insulin resistance in adipose tissue (88), and all of the adipokines in question could contribute to adipocyte (and systemic) insulin resistance. Impaired mitochondrial function induced by high iron is also a factor, possibly mediated by changes in heme oxygenase (89). Details of molecular mechanisms for the effects of iron on these and other factors are pleiotropic and discussed below.

In addition to the adipoldne-mediated remote effects of iron, iron also has direct effects of adipocyte metabolism, with one of the earliest reports showing that iron played a role in the antilipolytic effects of serum on adipocytes (90). Other pleiotropic effects of iron on intrinsic adipocyte metabolism have recently been reviewed (91).

#### 5.2.4. Low adipocyte iron protects from diabetes: Healthy obesity?

Perhaps even more impressive is the effect of low iron to protect from diabetes. This direct and causal antidiabetic effect of low adipocyte iron, as was suggested by the higher adiponectin levels in low-hepcidin states such as hemochromatosis, has also been demonstrated in animal models. Scherer’s group (92) has shown that overexpression of mitoNEET, an iron-sulfur cluster-binding protein in the outer mitochondrial membrane, lowers intracellular iron and mitochondrial iron transport. This results in increased adiponectin, decreased rates of fatty acid oxidation, massive obesity, and yet preserved insulin sensitivity. At least some of this salutary phenotype is direcdy linked to adiponectin insofar as it is ameliorated in adiponectin knockout mice and is consistent with the phenotype of mice overexpressing adiponectin (93). This healthy obesity phenotype was validated with transcriptional profiling that revealed a better vascularized, anti-inflammatory, and less-fibrotic environment in the fat with higher mitoNEET expression (94). These results also parallel the observation that low-serum ferritin in humans is a predictor of the lack of markers of metabolic syndrome (hypertension, dyslipidemia, and diabetes) despite extreme obesity (body mass index >40 kg/m^2^) (30).

Beneficial whole-body ramifications of low-adipocyte iron continue to emerge. Similar to the models discussed above, adipocyte-specific transferrin receptor 1 deficiency results in low-adipocyte iron and protects mice from high-fat-diet-induced metabolic disorders (80). This model reveals yet another mechanism, namely restricting lipid absorption from the intestine through modulation of vesicular transport in enterocytes following high-fat-diet feeding. The specific mediator of this effect is not yet understood but is among several examples of iron-regulated interorgan communication from adipocytes beyond the effects of iron on leptin and adiponectin levels. The mice described in Section 5.2.2 with overexpression of FtMT in adipose tissue, for example, develop robust beta cell hyperplasia (85). The same FtMT adipocytes also release small extracellular vesicles containing damaged mitochondria that are taken up by cardiomyocytes and release ROS, triggering upregulation of antioxidant defenses in the heart (95).

#### 5.2.5. Iron, adipocyte differentiation, and adipose tissue remodeling.

Consistent with its important role in regulating adipocyte function, adipocyte differentiation is also tied to the tight regulation of iron. Heme has long been known to promote adipocyte differentiation (96), but the simple availability of iron is not the entire story. Rather, preadipocytes are highly sensitive to an optimal level of iron, such that the effects of iron are described in a U-shaped curve. In cultured preadipocytes, both iron chelation and iron excess inhibited differentiation as well as the expression of both iron-related genes and genes involved in mitochondrial biogenesis (97). Interestingly, knockdown of one of the iron-related genes upregulated during differentiation, transferrin, inhibited differentiation. This illustrates the bidirectional interplay and interdependence between iron and metabolic regulation that exist in yeast and were considered above. Knockdown of transferrin in adipocytes also impairs insulin signaling (98). One mediator of this cross talk between iron and metabolism is cytosolic aconitase 1, which also functions as IRP-1 (99).

Adipose tissue thermogenesis has emerged as an important determinant of obesity and diabetes risk and is based largely on uncoupled mitochondrial fuel oxidation. Given the need for iron to metallate electron transport chain proteins and enzymes in that organelle, it is not surprising that iron regulation affects the development and differentiation of thermogenic (brown) fat and the remodeling of adipose tissue to become thermogenic (beiging). Iron-deficient mice (100) and humans (101) exhibit defective thermogenesis. Hypertrophy of brown adipose tissue in the iron-deficient mouse model and the acquired nature of the human iron deficiency suggest that these phenomena are functional (e.g., inadequate mitochondrial biogenesis or protein metallation) rather than developmental and are consistent with work by others on the relation of iron to brown adipose tissue function (102). The processes that link mitochondrial biogenesis with increases in iron uptake in adaptive or beiging thermogenesis are beginning to be understood and include modulation of the hypoxia-sensing pathway and hepcidin (103). At least some aspects of tissue remodeling in response to iron are specific to certain fat depots, with beiging in one obese mouse model being seen only in epididymal (visceral) fat (84).

#### 5.2.6. Importance of cross talk with adipose tissue macrophages.

Adipose tissue contains macrophages and vascular cells in addition to adipocytes. The role of macrophages in diabetogenesis is well appreciated, as is their role in iron homeostasis. Macrophages act as reservoirs of iron for other cell types, classically developing erythrocytes, and images of macrophages feeding erythroblasts are reminiscent of suckling piglets. Might the same relationship exist in other tissues? Recently, Hasty’s group (104,105) has accumulated evidence for just that, namely that adipose tissue macrophages are critical in determining the iron content of adipocytes. Specifically, in obesity or high-fat feeding, macrophages are activated and promote inflammation and insulin resistance. After high-fat feeding, macrophages also redistribute iron to adipocytes, a process possibly mediated by increased fatty acids (106).

The importance and physiological consequences of this iron redistribution are only beginning to be understood. One hint comes from a recent study of nondiabetic African Americans (78). Whole-body insulin resistance was found to be related to a genetic polymorphism resulting in reduced transferrin expression in adipocytes. This effect of the polymorphism was local and apparently limited to adipose tissue insofar as systemic transferrin levels were not affected. Supported by data from a cell knockdown model, the authors concluded that decreased transferrin resulted in decreased adipocyte iron, insulin resistance, and a transcriptional profile consistent with that phenotype. Those authors speculated that transferrin secreted by adipocytes participates in the paracrine trafficking of iron between macrophages and adipocytes, perhaps facilitating the adipocyte beiging that is observed with high-fat feeding.

Similar hypotheses have been advanced for iron trafficking between glial cells in the brain and neurons (107) and are being investigated for the epidemiologic associations between iron and numerous neurodegenerative diseases such as Alzheimer’s and Parkinson’s. Similar mechanisms may also be at play in other tissues such as liver, where macrophages and hepatocytes share responsibilities for iron homeostasis.

### 5.3. Iron and the Liver

Interactions among iron, diabetes, hepatic function, and hepatic damage present a complex picture. Features of metabolic syndrome have been linked to iron overload syndromes featuring the accumulation of iron in hepatic Kupffer cells, hyperferritinemia, relatively preserved hepcidin-mediated modulation of iron homeostasis, and normal circulating iron levels, a condition termed dysmetabolic iron overload syndrome (DIOS) (108–110). In DIOS, there is evidence for both insulin resistance contributing to iron overload and conversely iron overload contributing to insulin resistance, but the interactions among all of these factors cannot yet be fully disentangled. For example, iron is a risk factor for both diabetes and nonalcoholic fatty liver disease (NAFLD) (111, 112), and diabetes itself is also a common and major risk factor for NAFLD and cirrhosis. Conversely, cirrhosis is a risk factor for T2DM even when caused by an independent process such as viral hepatitis. This is illustrated in hemochromatosis, where iron is sufficient to cause cirrhosis without diabetes, and vice versa, but in those without hemochromatosis, the increased risk of diabetes seen with higher serum ferritin is accentuated in those individuals who also show evidence of hepatic damage (113). Thus, sorting out primary causality in these syndromes can be daunting. Mechanistic studies are beginning to elucidate the molecular pathways involved in these interactions, for example, transforming growth factor β (TGF-β) signaling in the iron-dependent progression of NAFLD (47). Thus, a better understanding of the multiple and complex relationships among insulin resistance, fatty liver, diabetes, and iron is proceeding. In the face of these many unknowns, this section concentrates specifically on the demonstrated effects of iron on insulin action and glucose metabolism.

Hepatic insulin resistance, at least for the control of hepatic glucose production, is a hallmark of T2DM. In the *db/db* (*lepr*^−/−^) mouse, iron overload aggravates insulin resistance and increases hepatic gluconeogenesis without evidence of increasing inflammation (114). Increased levels of Fe^2+^ induced by increasing heme oxygenase in liver likewise increase both insulin resistance and gluconeogenesis (115). Increased hepatic gluconeogenesis is also seen in a mouse model of hereditary hemochromatosis (42). Conversely, iron restriction causes hypoglycemia in a mouse model in part by decreasing hepatic pyruvate utilization for gluconeogenesis, driven by activation of the hypoxia sensing pathway (116).

A novel effect of iron is to alter the circadian rhythm of hepatic glucose production (117). Gluconeogenesis is normally suppressed during the usual feeding period (in humans during the day and in mice at night) and augmented during fasting. Disruption of this rhythm is associated with T2DM in experimental animal models and in humans, contributing to the diabetes risk seen in night shift workers (118). Although feeding, light/dark cycling, and glucoregulatory hormones all play roles, dietary iron also affects circadian gluconeogenesis. The effect of high dietary iron is to decrease the magnitude of the effects of circadian glucose metabolism through heme-mediated regulation of the interaction of nuclear receptor subfamily 1 group d member 1 (Rev-Erbα) with nuclear receptor corepressor 1 (NCOR). Increased heme is driven by upregulation of aminolevulinic acid synthase 1 mediated by peroxisome proliferator-activated receptor γ coactivator lα (PGC-lα).

Metformin exerts much or most of its antidiabetic action by inhibiting hepatic gluconeogenesis (119). Thus, a recent finding in yeast linking iron to metformin action may be relevant to understanding the mechanistic underpinnings of metformin action and the relation of iron to gluconeogenesis (14). The authors discovered a global cellular response to metformin that is similar to that of iron starvation with effects not only on glycolysis and glucose oxidation that mirror effects of metformin in human fiver, but also changes in DNA repair, activity of the mechanistic target of rapamycin pathway, and others that also are seen in higher organisms. Future work should clarify the relevance of this important finding to our understanding of iron and diabetes risk.

### 5.4. Iron and Muscle

Because of their high energy requirements, both skeletal and cardiac muscle are rich in mitochondria but at the same time also subject to oxidant stress from high rates of fuel oxidation. As a result, both high and low iron levels can impair mitochondrial function and contribute to poor exercise capacity, heart failure, and respiratory compromise, even within iron’s normal range and in the absence of anemia (reviewed in 120). In addition, low iron can decrease myoglobin levels, decreasing oxygen reserves for aerobic metabolism. Because skeletal muscle is the major site of insulin-stimulated glucose disposal, effects of disturbed iron homeostasis on glycemia might be expected, and mitochondrial dysfunction in muscle is a hallmark of T2DM and insulin resistance. Whether muscle mitochondrial dysfunction is a cause or consequence of insulin resistance is still controversial, although there is evidence that targeting mitochondria specifically can improve insulin action (121). Another possible connection of decreased muscle function to diabetes is production of the myokine IL-6 (122). Although usually associated with insulin resistance, IL-6 and other myoldnes produced after muscle contraction can have both inflammatory and anti-inflammatory systemic effects.

As discussed in Section 4, high iron states are associated with hypermetabolism, and this is manifest in muscle as evidenced by increased rates of fatty acid oxidation mediated by AMP-dependent kinase (42,123). This is associated with decreased adiposity, which although protective of diabetes by itself, is overridden by loss of insulin secretion, resulting in diseases such as HH that are associated with high diabetes risk.

### 5.5. Iron and the Microbiome

The gut microbiome has emerged as a significant contributor to the risk of diabetes and obesity (124). Iron has significant effects on the composition of the gut microbiome in ways that influence the risk of both T2DM and fatty liver (125,126). As is true of other connections between iron and diabetes, the effects are bidirectional, with iron modifying the microbiome and the microbiome, in turn, modifying iron regulatory mechanisms in the host.

## 6. MOLECULAR SIGNALING MECHANISMS FOR THE EFFECTS OF IRON ON METABOLIC REGULATION AND DIABETES RISK

In many of the cases considered above, there is good evidence for how iron confers diabetes risk, for example, mediation by endocrine factors such as adiponectin. In this section, we examine the more proximal intracellular signaling mechanisms triggered by iron. Like many of the tissue effects reported above, and given the fundamental need for iron and its participation in so many pathways, there is no single mechanism. Nevertheless, the mechanisms that have been dissected do fall into categories, and unifying principles have emerged. Furthermore, the pathways are not mutually exclusive and in some cases are even directly linked to one another (e.g., oxidant stress with both hypoxia and inflammation). The literature dealing with these pathways is large, and the length restrictions of publication allow us to cite only representative references and require us to concentrate on pathways with the most definitive evidence suggesting causal relationships. Other potential pathways with less conclusive mechanistic evidence [e.g., the AMP-dependent kinase (AMPK)-sirtuin pathway (123)], however, deserve further study.

### 6.1. Oxidative Stress

Through Fenton chemistry, ferrous(II) iron can interact with hydrogen peroxide to generate ferric(III) iron and hydroxyl radicals. Resultant ferric iron can also react with peroxide to generate perhydroxyl radicals, and both are capable of oxidizing intracellular lipid, protein, and DNA. Resultant lipid peroxidation can result in further damage through pathways such as programmed cell death through ferroptosis (127). Pancreatic beta cells have received particular attention in terms of oxidative stress and iron, not only because of their key role in diabetes but also the fine tuning of their redox systems that is necessary for normal glucose-induced insulin secretion (see Section 5.1 and 48). One of the principal antioxidant-protective enzymes in pancreatic beta cells is GPx4, and its activity in reducing lipid peroxides plays a particular role in protection from ferroptosis in those cells. Thus, both overexpression of GPx4 (49) and inhibition of ferroptosis (56) can rescue islets from stress induced by high iron and high glucose. These may play a particularly important role in T2DM and iron overload, wherein GSH (oxidized in the GPx4 reaction) and GPx4 are diminished.

Many other oxidant stress-driven pathways other than ferroptosis are linked to diabetes pathogenesis, and iron as a generic oxidant can therefore be plausibly linked to any or all of them, including endoplasmic reticulum stress. Specific mechanistic studies that directly implicate iron in such processes have been performed and generally support the role of iron in their activation. Iron, for example, stimulates clustering of the endoplasmic reticulum stress activator Irel (128). Detailed mechanisms underlying such effects may be much more nuanced than the generation of oxidant species. For example, yeast mutant library screening to elucidate mediators suggests that heme-dependent sterol synthesis may be the pathway through which this effect proceeds. In other cases, however, the effects may be more direcdy related to the generation of oxidants. In adipose tissue, for example, iron chelation results in decreased superoxide production, macrophage infiltration of fat, and inflammatory cytokine production in a mouse T2DM model (129). But even in cases such as this, the intermediate pathways induced by iron, both protective and pathogenic, are complex, with multiple proteins such as heme oxygenase playing roles in processes such as iron-induced mitochondrial dysfunction in adipocytes (89).

### 6.2. Inflammation/Cytokines

The upregulation of inflammatory cytokines by iron was considered above (Section 5.2), but given the protean effects of activating inflammation pathways on insulin signaling and other diabetes-related pathways, those findings likely represent the tip of the iceberg. The reader is directed to targeted reviews of these processes for more in-depth information (e.g., 130).

### 6.3. Hypoxia Sensing

One attractive candidate for mediating the effects of iron on diabetes risk is the hypoxia-sensing pathway, given its iron and oxygen dependency and its functions in adjusting metabolism in response to hypoxia by augmenting glycolysis (the Warburg effect), thus decreasing glucose and fatty acid oxidation and activating antioxidant defenses (131). There is an inverse association between resident altitude, activation of the pathway, and diabetes risk (132, 133). Hypoxia and iron deficiency inactivate prolyl hydroxylase domain (PHD) proteins and an asparaginyl hydroxylase [factor inhibiting hypoxia (FIH)], leading to transcriptional activation by hypoxia-inducible factors (HIFs) through increasing their stability and interactions with the transcriptional coactivator cAMP response element-binding protein (CREB) binding protein, p300 (131,134). Although both hydroxylases require iron, FIH is strikingly more sensitive to deactivation by oxidant stress (135), so the pathway can also be activated by ROS resulting from high iron. Among the many effects of HIFs on transcription, they also activate iron uptake pathways in concert with their effects to increase hemoglobin concentrations in hypoxia.

Although the hypoxia pathway plays roles in many pathways potentially linked to the role of iron in diabetes risk [adipose dysfunction (136), regulation of fatty acid oxidation (131), and stimulation of glucagon-like peptide-1, GLP-1 (137)], its specific link to iron levels has only been directly investigated in a few systems. In the case of adipocyte dysfunction, iron chelation has been shown to improve insulin action and hypoxia pathway signaling and to decrease levels of inflammatory mediators in adipocytes of an obese diabetic mouse model, although direct causality of the hypoxia pathway was not demonstrated (87). The same is true of a study demonstrating improved insulin sensitivity in mouse liver with iron chelation (13 8). In a study of another process contributing to diabetes, hepatic glucose production, it was shown that hypoxia, and more so hypoxia with iron deficiency, resulted in hypoglycemia and impaired hepatic gluconeogenesis (116), although the prodiabetic converse of high iron and normoxia was not studied. Thus, although the HIF pathway remains one of the most plausible mediators of the intersection of iron and diabetes risk, its role remains understudied.

### 6.4. Mitochondrial Dysfunction

Mitochondrial dysfunction is a hallmark of diabetes, and inherited mitochondrial diseases usually include diabetes among their manifestations, including Friedreich’s ataxia that is characterized by iron overload (139). A diabetic mouse model was protected by iron restriction, exhibiting improved mitochondrial function and insulin secretion (26). Mitochondrial dysfunction with impaired insulin secretory capacity and defective glucose-stimulated insulin secretion were also seen in an HH mouse model (140) and in other models described in the section describing the protean contributions of adipocytes to iron-related phenotypes. Interestingly, in the HH model, iron overload was manifest in the cytosol but not mitochondria, where levels of other divalent cations including Mn^++^ were low (140, 141). Mn^++^ supplementation improved insulin secretion, activity of the antioxidant Mn superoxide dismutase, and oxidant stress, suggesting a novel mechanism of iron toxicity through impairment of the trafficking of other metals that participate in antioxidant defense mechanisms and may share transport or chaperone proteins with iron.

### 6.5. Transforming Growth Factor-β

TGF-β is an interesting mediator of prodiabetic iron effects insofar as it is a member of a superfamily of growth factors that includes the bone morphogenetic proteins that also regulate iron (142). TGF-β triggers collagen production in hepatic stellate cells, and deletion of the TGF-β receptor in hepatocytes alleviates NASH (143). TGF-β and signaling partners in the SMAD pathway are increased in mice on a high-iron diet that also accelerated NASH (47, 144). Its fall after iron restriction in a diabetic rat model has also been tied to improvements in diabetes and insulin production (1). Interestingly, TGF-β is also upregulated by glucose and is a mediator of diabetic nephropathy (145).

### 6.6. Transcription

The multiplicity of pathways affected by iron is no more evident than in the studies that have addressed transcriptional pathways affected by iron. These are well illustrated by the effects of iron on leptin, adiponectin, and the circadian rhythm. In the cases of leptin and adiponectin, promoter mutagenesis and chromatin immunoprecipitation identified the transcription factors responsible to be CREB and forkhead box Ol (FOXOl), respectively (30, 146). In the case of the circadian rhythm, PGC-lα proved to play a central role in stimulating the association of Rev-Erbα with its corepressor complex (117). The peroxisome proliferator-activated receptors have been implicated in iron effects in beta cell function (1). Two points are noteworthy. First, this collection of proteins includes some of the factors most central to metabolic regulation: They coordinate the fasting/fed transition and participate in mitochondrial biogenesis, adipogenesis, and lipid sensing. Second, they have relatively litde in common with regard to structure or regulatory mechanisms.

### 6.7. Pbsttranslational Modification as a Possible Unifying Mechanism

The diversity of mediators of the effects of iron may be informative. Might it suggest there could be an upstream, common regulatory mechanism involved, a mechanism that can regulate this set of effectors? This has been most extensively studied in the case of leptin and CREB, wherein increased CREB phosphorylation led to increased CREB occupancy of inhibitory sites despite the fact that the usual CREB kinases were not affected by iron (74). It was previously determined that the pathway of hexosamine biosynthesis and protein modification by O-linked A/-acetylglucosamine (O-GlcNAc) was involved in the regulation of leptin (147, 148), which spurred investigation into the role of O-glycosylation in iron-mediated regulation of leptin (74). The studies determined that iron downregulates leptin by decreasing CREB glycosylation, resulting in increased CREB phosphorylation and occupancy of the leptin promoter.

O-GlcNAcylation as an integrating principle in iron regulation is attractive on many fronts. This modification of proteins is quantitatively as common as phosphorylation and is highly dynamic (149). The modification is controlled through the activities of a single O-GlcNAc transferase and a single enzyme that removes O-GlcNAc. Signaling by O-GlcNAcylation of proteins serves as an integrative, nutrient-responsive regulatory system, rate limited by hexosamine synthesis. O-GlcNAc modification is often reciprocal to phosphorylation, in some cases because of shared or proximate modification sites. Thus, O-GlcNAc serves in part as a nutrient-based modulator of phosphate-mediated signaling. Importantly, it affects the activity of other transcription factors including PGC-lα (150), FOXOl (151), HIFs (152), and the peroxisome proliferator-activated receptors (153). Furthermore, O-GlcNAcylation is involved in the oxidative stress response (154). TGF-β, another mediator of the effects of iron on diabetes risk mentioned above, is also regulated by the hexosamine pathway (155).

## 7. CONCLUSIONS AND FUTURE DIRECTIONS

Iron, given its requirement in numerous metabolic pathways but simultaneous danger as an oxidant, is highly regulated. It is therefore not at all surprising that iron availability and metabolism are tighdy coupled, and that both iron deficiency and excess are accompanied by broad changes in metabolism, including increased risk of diabetes. These changes are manifest in multiple tissues and through multiple mechanisms that are still incompletely understood. The role of the brain in regulating metabolism is becoming increasingly recognized, and brain iron homeostasis is incompletely understood: Why, for example, is hypogonadotropic hypogonadism a cardinal complication of hemochromatosis? And with the multiplicity of mechanisms underlying the effects of iron on different tissues, are there commonalities in the upstream signaling for those changes?

Because normal iron levels are defined as those that do not lead to overt pathology, it is likely that there are optimal levels of iron in a much narrower range than the population norms. Further studies are needed to define that optimal range and the underlying mechanisms for the protean effects of iron so that we can better fine-tune iron levels to improve metabolic health.

## SUMMARY POINTS

1. Iron absorption and availability are tighdy regulated through multiple mechanisms due to iron’s requirement for redox reactions used in fuel metabolism but its simultaneous danger as a potent oxidant.
2. Iron is an established risk factor for diabetes in pathologic iron overload, and recent data demonstrate this risk is also evident in the broad range of normal iron.
3. Consistent with that risk, iron affects the major determinants of glucose homeostasis (insulin secretory capacity, insulin sensitivity, and hepatic glucose production) in the tissues that determine homeostasis.
4. In the pancreatic beta cell, both deficient and excess iron contribute to decreased insulin secretion and coupling of blood glucose levels to insulin secretion.
5. In the adipocyte, high iron alters fat metabolism and decreases both the level of the insulin-sensitizing hormone adiponectin and the appetite-regulating hormone leptin.
6. In the liver, high iron regulates changes in hepatic glucose production and insulin sensitivity.
7. A multiplicity of molecular mechanisms for these effects argues for the existence of a higher-order mechanism that integrates iron availability into the processes that determine metabolic activity, fuel choice, and other key determinants of diabetes risk.

## Abbreviations

T2DM: type 2 diabetes mellitus
IRP: iron regulatory protein
NASH: nonalcoholic steatohepatitis
ROS: reactive oxygen species
GPx4: glutathione peroxidase 4
DMT1: divalent metal transporter 1
IL-6: interleukin 6
TNF-α: tumor necrosis factor α
NAFLD: nonalcoholic fatty liver disease
TGF-β: transforming growth factor β
Rev-Erbα: nuclear receptor subfamily 1 group d member 1
NCOR: nuclear receptor corepressor 1
PGC-lα: peroxisome prohferator-activated receptor γ coactivator lα
PHD: prolyl hydroxylase domain
ETH: factor inhibiting hypoxia
HIF: hypoxia-inducible factor
CREB: cAMP response element-binding protein
GLP-1: glucagon-like peptide-1
O-GlcNAc: O-linked *N*-acetylglucosamine
